# Plasmon-induced hot electron transfer in AgNW@TiO_2_@AuNPs nanostructures

**DOI:** 10.1038/s41598-018-32510-2

**Published:** 2018-09-20

**Authors:** Jiaji Cheng, Yiwen Li, Marie Plissonneau, Jiagen Li, Junzi Li, Rui Chen, Zikang Tang, Lauriane Pautrot-d’Alençon, Tingchao He, Mona Tréguer-Delapierre, Marie-Hélène Delville

**Affiliations:** 1 CNRS, Univ. Bordeaux, ICMCB, UMR 5026, F-33608 Pessac, France; 2 College of Physics and Energy, Shenzhen University, Shenzhen, 518060 People’s Republic of China; 3 Department of Electrical and Electronic Engineering, Southern University of Science and Technology, Shenzhen, 518055 China; 4 The Institute of Applied Physics and Materials Engineering, University of Macau, Avenida da Universidade, Taipa, Macau China; 5 School of Science and Engineering, The Chinese University of Hong Kong, Shenzhen, People’s Republic of China; 6 Solvay, 52 rue de la Haie Coq, Aubervilliers, F93308 France

## Abstract

Compared to the limited absorption cross-section of conventional photoactive TiO_2_ nanoparticles (NPs), plasmonic metallic nanoparticles can efficiently convert photons from an extended spectrum range into energetic carriers because of the localized surface plasmon resonance (LSPR). Using these metal oxide semiconductors as shells for plasmonic nanoparticles (PNPs) that absorb visible light could extend their applications. The photophysics of such systems is performed using transient absorption measurements and steady extinction simulations and shows that the plasmonic energy transfer from the AgNWs core to the TiO_2_ shell results from a hot carrier injection process. Lifetimes obtained from photobleaching decay dynamics suggest that (i) the presence of gold nanoparticles (AuNPs) in AgNWs@TiO_2_@AuNPs systems can further promote the hot carrier transfer process via plasmonic coupling effects and (ii) the carrier dynamics is greatly affected by the shell thickness of TiO_2_. This result points out a definite direction to design appropriate nanostructures with tunable charge transfer processes toward photo-induced energy conversion applications.

## Introduction

Photo-driven chemical reactions, such as solar water splitting^1,2^, photodegradation^3^, solar conversion to electricity for photodetector^4^, solar cells^5^ and ultrafast optical data storage^6,7^ are widely considered for future applications towards renewable energy resources^8–12^. The underlying principle of photo-induced energy transformation is mainly based on the formation and separation of electron-hole pairs in semiconductor materials^13,14^. However, the limited absorption cross-section and narrow spectrum of conventional photoactive semiconductors (such as TiO_2_) due to their relatively large band gaps, could impede their further development for commercial value. Over the past decade, plasmonic metallic nanoparticles were extensively scrutinized due to their fascinating optical properties through an excitation of surface plasmon resonance (SPR)^15^. Surface plasmon is an incompressible electron cloud around finite metal, which exhibits a light-induced collective oscillation of these free electrons with incident photons as long as the collective coherent oscillation matches the frequency of photons. When localized at the surface of metallic nanoparticles this light-excited collective electron charge oscillation allows new strategies to provide platforms for conventional semiconductors, susceptible to cover the entire solar spectrum. Indeed, the tunability of the plasmon wavelength can be accurately controlled through the metallic nanoparticles properties such as their size, shape, and chemical composition^16^. The highly active photo-induced hot carriers (electrons with energies higher than the metal Fermi level) located on the metal are assumed to transfer to the conduction band of the semiconductor through a plasmon decay provided desirable nanostructures contact is achieved, leaving a “hot” hole on the metal^17^.

However, the transfer of hot carrier from metal to semiconductor derived from the surface plasmons decay might take place through multiple channels, and detailed mechanisms are still to be fully understood^18^. In brief, the transfer channels are categorized into either a radiative process, which could combine with far-field scattering and near-field electromagnetic enhancement effect, or a non-radiative process via SPR-mediated hot carrier injection^10,19^. In order to thoroughly investigate these mechanisms, it is necessary to elucidate the plasmon photo-physics process at various timescales, relating the energetic carrier dynamics and the transfer. The ultrafast measurement is a powerful approach to understand the dynamics of photo-induced electrons and holes in hetero-nanostructures^20,21^. The ultrafast dynamics of pure plasmonic metallic NPs have been investigated for more than two decades^22,23^. Many authors focused on nanospheres, nanorods, nanoprisms under transient absorption spectroscopy (TAS) measurements^24–26^, and better clarified the time scale of electron-phonon coupling and phonon-phonon coupling according to a two-temperature model^27,28^, and such a technique has even been extended to novel plasmonic oxides such as indium tin oxide (ITO) nanowires^29^ and indium-doped cadmium oxide (ICO) nanocrystals^30^. Nevertheless, compared with individual metals or semiconductor materials, hybrid systems, typically plasmon-semiconductor nanostructures, have rarely been reported so far. To date, M. Sun *et al*., for example, have investigated the plasmon-exciton interactions of metal and semiconductor hybrids for surface catalytic reactions^31–34^ via ultrafast pump-probe TAS in the Vis-NIR region. They demonstrated the probability and the enhanced efficiency of the surface Raman scattering of these hybrids to be co-driven by graphene-AgNWs hybridization^35^. Further, N. Wu and co-workers studied metal@Cu_2_O interactions such as Ag@Cu_2_O, Au@Cu_2_O and Au@SiO_2_@Cu_2_O core-shell nanostructures via TAS and showed that the photocatalytic activities were raised by simultaneously a hot electron transfer (HET) and a plasmon-induced resonant energy transfer (PIRET)^36–38^. These works demonstrate that (i) plasmon mediated charge and energy transfer can overcome the band edge constraints of single semiconductors, and (ii) the metal-semiconductor core-shell interactions have promising potentials for enhancing solar-light harvesting and energy-conversion efficiency. However, detailed explanations on the mechanisms concerning how metal-semiconductor interactions take place, and a universally applicable physical modelling considering size, shape and compositions of the hybrids still remain challenging and need more experimental and theoretical supports^11^.

**Figure 1:**
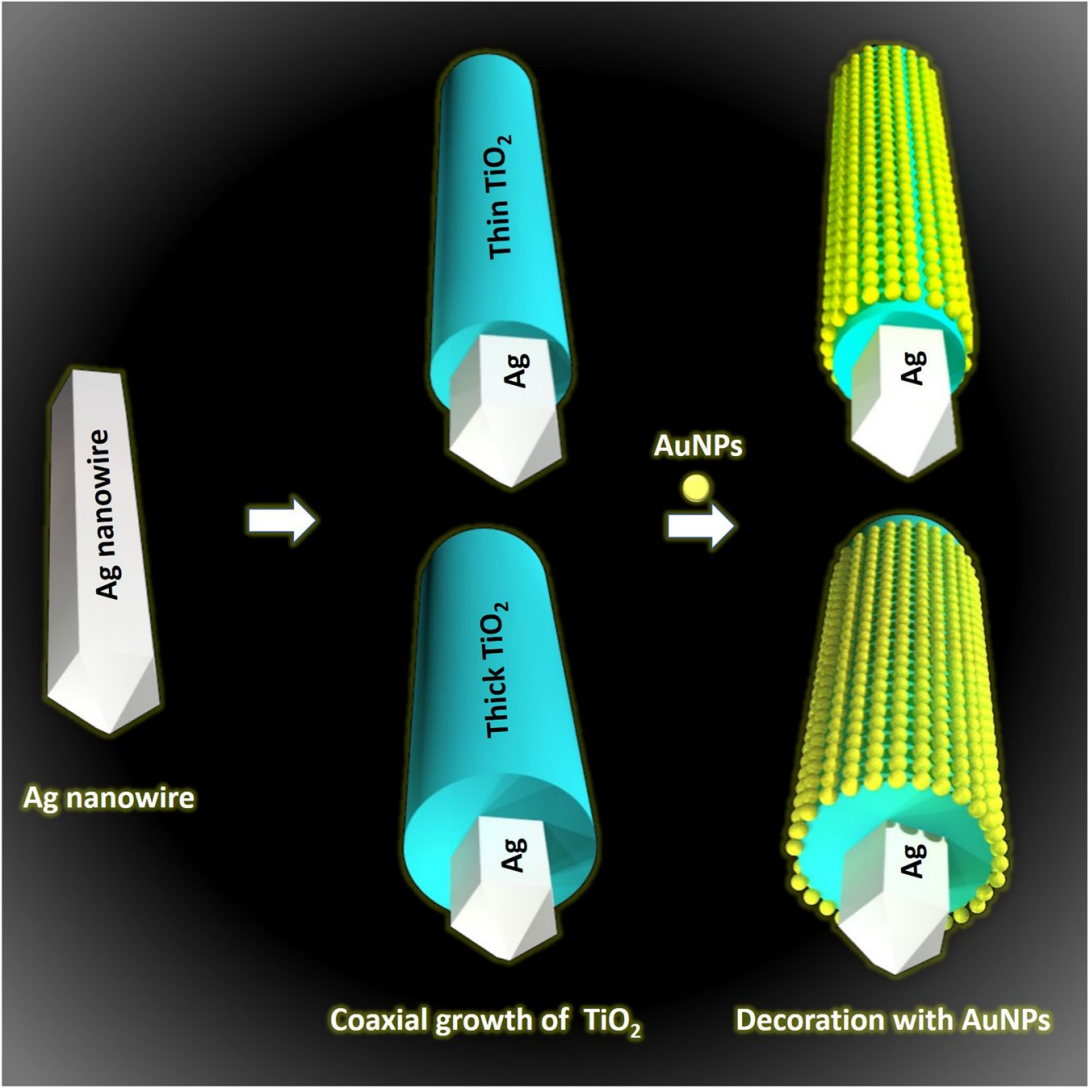
Schematic representation synthesis of the AgNWs@TiO_2_@AuNPs heterostructures with thin and thick layers of TiO_2_. White pentagonal prism stands for Ag, blue coating stands for TiO_2_ layer, and light-yellow particles are AuNPs.

Herein, in the present work, we prepared AgNWs@TiO_2_@AuNPs heterostructures through a facile wet chemical approach. As illustrated in Fig. 1, the as-synthesized AgNWs (depicted as the white pentagonal prism) are firstly coated with a TiO_2_ shell with well-defined thickness (blue) via typical sol-gel chemistry, and then such nanostructures are further grafted with AuNPs (yellow) to obtain AgNWs@TiO_2_@AuNPs heterostructures. These tunable nanostructures allowed focusing on the plasmon decay lifetime observation under the influence of various parameters. We finally studied two effects: (i) TiO_2_ shell thickness vs. silver nanowires photobleaching lifetime resulted from plasmon mode. (ii) The emerging transfer channel raised by AuNPs decorated on the TiO_2_ surface. These new observations may provide valuable strategies to design better SPR-mediated carrier transfer nanostructures towards future promising photochemical transformation devices.

## Results and Discussion

### Morphology and spectral properties of plasmonic TiO2 nanostructures

PVP (poly(vinyl pyrrolidone)) coated silver nanowires (40 ± 2 nm) were functionalized via MUA (mercaptoundecanoic acid) in pure ethanol. Then, a thickness-controlled layer of titania was deposited on the surface of AgNWs by using a proper sol-gel chemistry. Since it was well-known that the reactivity of titanium alkoxides is much higher than that of silicon alkoxides, e.g. TEOS the hydrolysis-condensation rate of the titanium precursor, i.e. TTIP (titanium (IV) tetraisopropoxide), needed to be slowed down via chemical retardants. Acetyl acetone (Acac) is such a complexing ligand which by coordinating titanium makes the Ti-Acac bond much less hydrolysable^39^. The reactivity of TTIP is then generally controlled through the molar hydrolysis ratio: H_2_O/Ti and the complexation ratio: Acac/Ti. Basically, when Acac/Ti ≥ 2, the resulting complex is stable enough to slow down the hydrolysis of TTIP for coating on AgNWs. (Data not shown here).

**Figure 2:**
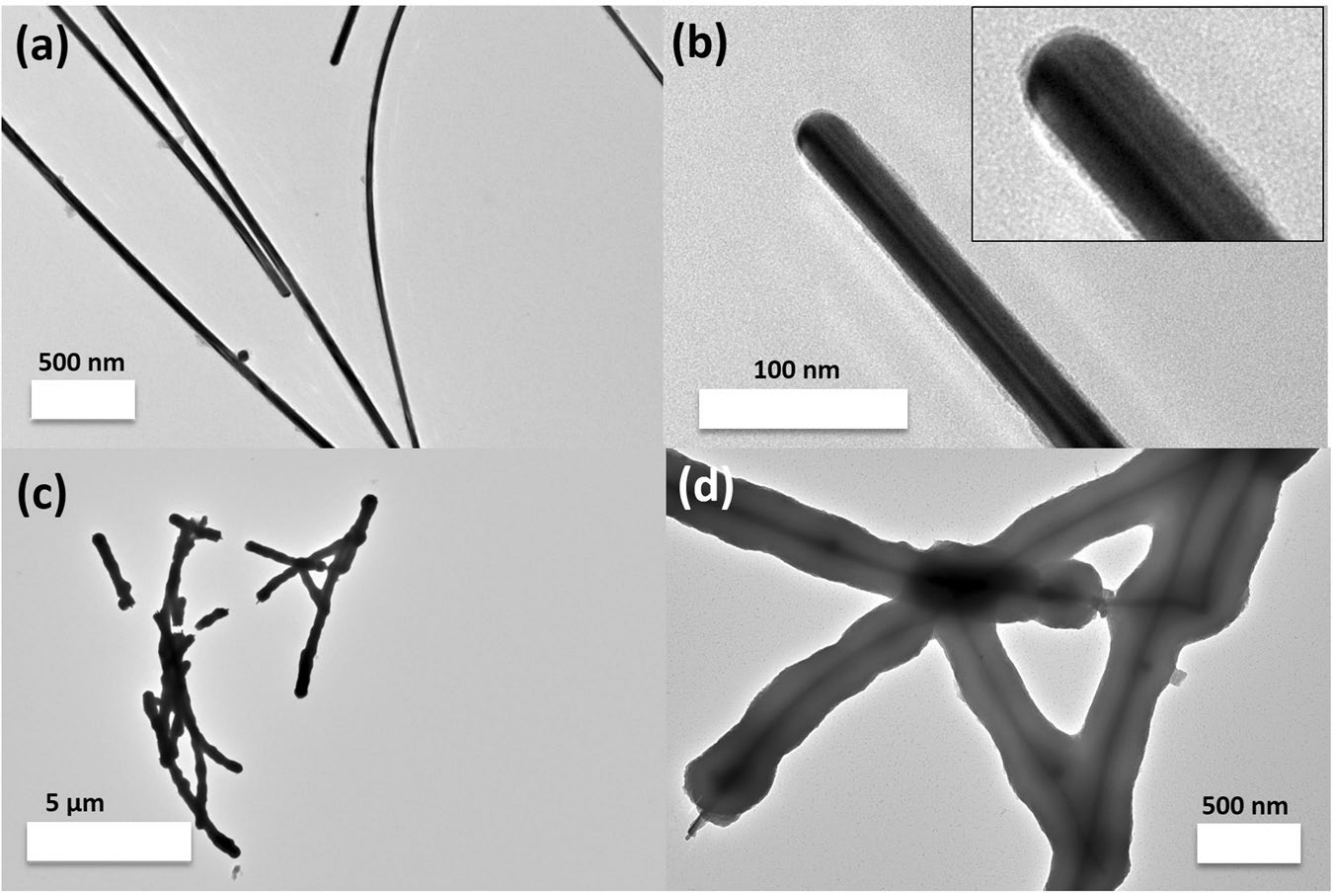
TEM images of AgNWs@TiO_2_ nanostructures synthesized with (**a,b**) H_2_O/Ti = 1.5, (**c,d**) H_2_O/Ti = 4.5. Inserts are magnified TEM image. The estimated thickness of TiO_2_ coating in (**b**) is 1.8 ± 0.3 nm and in (**d**) is 140 ± 9 nm. All the statistics are performed with more than 100 times measurements of the objects. 1 mL 14 mM TTIP mixed with Acac (1:2 molar ratio) was prepared for each run.

Then, the molar hydrolysis ratio of H_2_O/Ti and the total concentration of Ti precursor are crucial for the thickness-controlled coating of TiO_2_ onto the AgNWs. As an example, at a fixed concentration of 14 mM TTIP (Acac/Ti ratio: 2/1), when H_2_O/Ti ≤ 1.5, almost no clear coating of TiO_2_ (1.8 ± 0.3 nm) is observed indicating that the hydrolysis-polycondensation process was mainly inhibited probably because there was not enough water (Fig. 2a,b). When H_2_O/Ti = 4.5, a thick layer (140 ± 9 nm) of TiO_2_ and no free titania nanoparticles were observed (Fig. 2c,d), which suggests that at this molar hydrolysis ratio, the TTIP could be fully hydrolyzed and polycondensed on the AgNWs surface.

**Figure 3:**
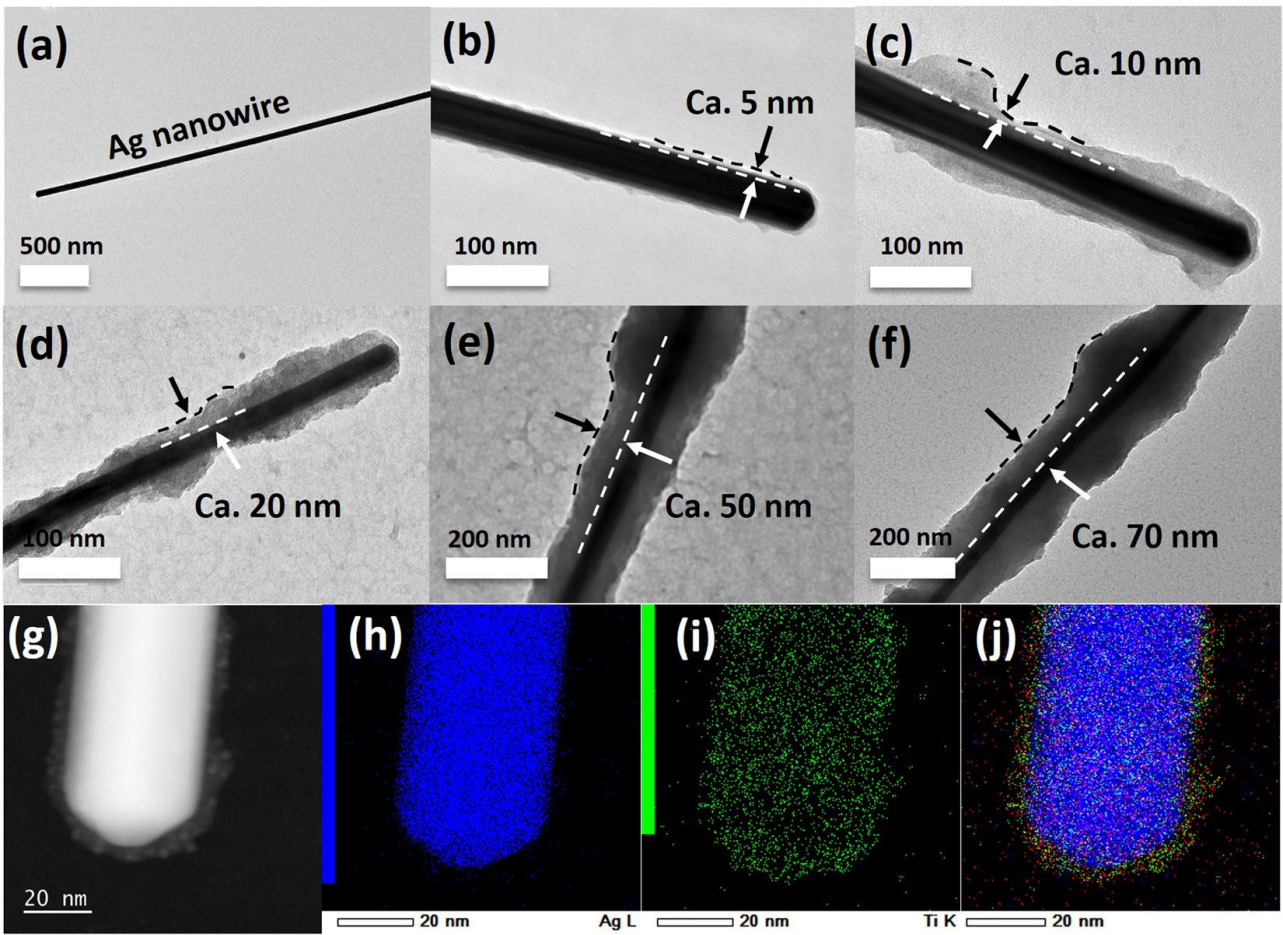
Controlled synthesis of AgNWs@TiO_2_ core-shell nanostructures. (**a**) typical image of a silver nanowire; (**b**–**f**): typical TEM images showing the variation of the average thickness of the TiO_2_ shell as a function of the concentration of TTIP precursor (from 5 to 70 nm, with TTIP concentration from 1.4 mM to 7 mM, respectively). More than 50 measurements were done for each sample for the thickness estimation and circa values were obtained by rounding off; (**g–j**) Dark field TEM image (**g**) of a AgNWs@TiO_2_ nano-object with a 5 nm shell thickness as in (**b**) and its corresponding EDX-mapping of (**h**) Ag, (**i**) Ti and (**j**) overlay of Ag, Ti and O.

**Figure 4:**
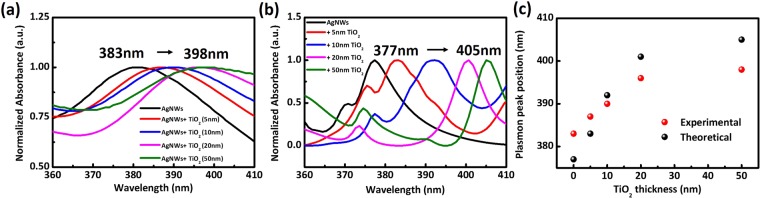
(**a**) Effects of titania thickness on the UV spectra of titania-coated silver colloids. There is a red-shift of the peak position with increasing TiO_2_ thickness. (**b**) Calculated extinction spectra of Ag nanowires without and with different TiO_2_ shell thicknesses via FDTD method. The small shoulders are the higher ordered multipole effects. (**c**) Plot of transverse plasmon peak position of AgNWs@TiO_2_ vs. the TiO_2_ shell thickness: black dots: theoretical results and red dots: experimental ones. All AgNWs have a diameter of 40 ± 2 nm and lengths of tens of µm with narrow size distribution.

In order to obtain a tunable thickness of the TiO_2_ coating down to several nanometers, the concentration of the Ti precursor was then studied. To investigate this effect, the H_2_O/Ti ratio was set as 4.5 to make sure all the Ti precursor was hydrolysed as previously indicated, and the Ti precursor solution (14 mM TTIP, Acac/Ti = 2) was diluted 2 to 10 times as described in the experimental part. In this way, the thickness of the TiO_2_ coating was strongly related to the concentration of TTIP as shown in Fig. 3. When the initial TTIP concentration is increased from 1.4 mM (ten times dilution) to 7 mM (two times dilution) and 14 mM (no dilution), the thickness of TiO_2_ coating varied from approximately 5 nm, to 70 nm and 140 nm respectively as confirmed by TEM observations (Fig. 3b–f) and EDX-mapping (Fig. 3g–j) of the sample presented in Fig. 2b. The absorption spectra of the silver sols change during the deposition process, as shown in Fig. 4a, where the position of the surface plasmon band is shown to be dependent on the titania thickness. The peak position prior to titania deposition was 383 nm; it red-shifted for silver sols modified with an increasing amount of titania. The red-shift is caused by the increased refractive index around the colloid particles after the titania deposition and the scattering from the shell and the thicker the semiconductor coating around the particles, the larger the shifts. For a shell thickness beyond 70 nm, almost no clear plasmon band was observed (Data not shown), indicating that a sufficiently large titania shell promotes significant scattering which makes the plasmon band of AgNWs hard to be detected. Theoretical extinction spectra were calculated to quantify the effects of the different titania shell thicknesses on the optical response of the silver nanowires (Fig. 4b)^40^. The simulation considered model nanowires with a pentagonal symmetry coated with uniform titania shell. Johnson and Christy’s data^41^ were used for the silver dielectric function, while a uniform refractive index of 1.33 was used for the nanoparticle environment as this value typically leads to a good reproduction of resonance positions of chemically synthesized nano-objects in aqueous suspension with noting that the small shoulders are the higher ordered multipole effects such as quadrupole. These simulations do confirm that the main effects of increasing the thickness of the titania layer are a red-shift and a broadening of the plasmon resonances. Figure 4c shows the position of the extinction peak predicted through calculations using the Finite-Difference Time-Domain (FDTD) method to calculate the fields and the associated absorption and scattering cross-sections., which were basically in qualitative agreement with the measurements.

**Figure 5:**
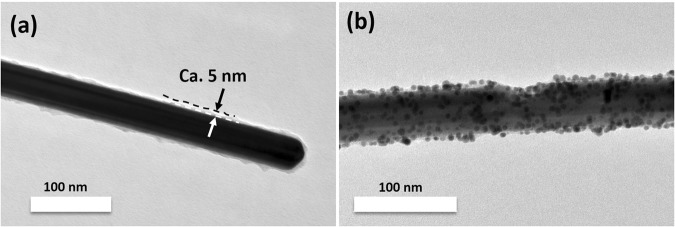
Typical TEM images of AgNWs@TiO_2_ nanostructures with 5 nm thickness of TiO_2_ shell before and after AuNPs grafting.

AgNWs@TiO_2_@AuNPs hybrid nanostructures were synthesized thanks to electrostatic interactions acting as the driving force between negatively charged AgNWs@TiO_2_ nanowires in pure water (IEP_TiO2_ close to 6.2) and the positively charged APTES (APTES: 3-Aminopropyl) triethoxysilane)-modified gold nanoparticles introduced at pH~8. A typical grafting of the AuNPs on the AgNWs@TiO_2_ nanowires with 5 nm TiO_2_ shell is illustrated in Fig. 5. The overall surface of the AgNWs@TiO_2_ can be tagged with gold nanoseeds (4–5 nm). Further, as the TiO_2_ thickness increased from 5 nm to 20 nm, a red-shifted from 383 nm to 400 nm of the characteristic transverse SPR mode of the AgNWs is observed and a weak absorption peak (except in the 20 nm TiO_2_ sample) at 538 nm which stands for the absorption of the grafted AuNPs on the AgNWs@TiO_2_ surface is generated (Fig. S2 and Table 1). These observations are probably due to both the effects of refractive index increase and the plasmonic coupling of AuNPs-AgNWs and closely packed AuNPs (the AuNPs alone have a SPR peak at 519 nm, see Fig. S3), since the size of grafted AuNPs does not change and no clear agglomerates form during the synthesis. Notably, the same amounts of AuNPs were used in each AgNWs@TiO_2_@AuNPs synthesis suggesting that for 10 nm and 20 nm TiO_2_ samples, the grafting of AuNPs would be less efficient than for the thinner 5 nm TiO_2_ coating sample due to the increased surface area, which leads to a less densely packed surface of AuNPs (Fig. S2).

**Table 1:** Summary of the UV absorption peak positions of AgNWs@TiO_2_@AuNPs nanostructures.

Sample	Peak positions (nm)		
AgNWs	356	383	
AgNWs + TiO_2_ 5 nm + AuNPs	357	385	538
AgNWs + TiO_2_ 10 nm + AuNPs	357	386	538
AgNWs + TiO_2_ 20 nm + AuNPs	358	400	—

### Non-radiative carrier relaxation dynamics of AgNWs@TiO_2_

In metallic nanoparticles, the coherent plasmon oscillation induced relaxation dynamics can undergo electron-electron scattering followed by electron-phonon scattering processes^27^. Time-resolved pump-probe spectroscopy measurement is then a powerful tool to examine these processes due to their desirable time scale down to the picosecond. The transient absorption contour map of pure AgNWs is displayed in Fig. S4a. The photo-bleaching signal evidences the decay of the plasmon-induced electron relaxation due to the coincidence of the bleaching and the steady-state absorption peaks. The bleaching maxima locates around 349 nm and 371 nm resulting from the plasmonic modes of the pentagonal silver nanowires similar to those of bulk Ag at 350 nm^42–46^. Initially, the nanowires produce a non-Fermi electron distribution after the dephasing resulting from elastic and inelastic electron scattering (about 10 fs). The ultrafast LSPR dephasing time is usually estimated through the line widths measurements of single nanoparticles. The distribution can quickly bring thermalization *via* electron-electron scattering, which results in a high temperature electron distribution. After this process, the electron gas, which has plasmon energy cools down to equilibrate the temperature between electron and lattice, which is attributed to electron-phonon interactions. These established photo-physics mechanisms explain the bleaching peak around the plasmon wavelength and the subsequent decay of bleaching over a period of time within picoseconds. The corresponding electron-phonon coupling time τ_e_-ph can be calculated as γT_0_/G under low excitation powers, where T_0_ is the ambient temperature and γ is a damping constant^27,47^. The corresponding electron-phonon coupling constant G directly determines the electron relaxation lifetime; the stronger the interaction between electrons gas and phonon in the lattice and the faster the electron gas cooling as well as the shorter the bleaching decay in TAS spectra^27^.

**Figure 6:**
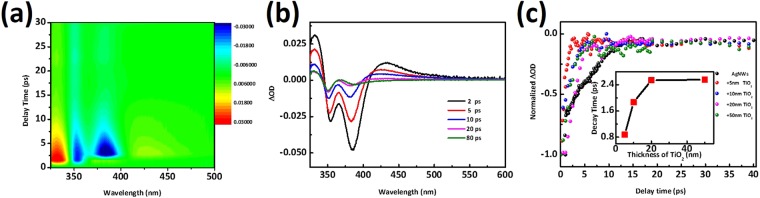
Transient absorption measurements of the AgNWs with different TiO_2_ shell thicknesses. (**a**) ΔOD spectral mapping of AgNWs@TiO_2_ with a 20 nm shell. (**b**) Transient absorption spectra at various time delays of AgNWs@TiO_2_ with 20 nm shell (**c**) Normalized curves of shell-thickness dependence of the AgNWs plasmon photobleaching decay dynamics. Probe wavelength is 349 nm. All the experiments were conducted under pump wavelength of 320 nm (3.87 eV) with average power of 3 mW.

Time-resolved pump-probe spectroscopy measurements of the core-shell nanostructures are illustrated in Figs 6a and S4a, which show the TAS 2D color map of AgNWs with 20 nm TiO_2_ shell as well as that of the pure AgNWs, respectively. We select 3.87 eV pump beam energy for the plasmon excitation in order to avoid the pump scatter disturbance or saturation onto the plasmonic peak (at 350 nm)^48^. The differential transmission at various time delays of the same sample (Fig. 6b) shows two bleaching peaks located at 357 nm and 388 nm resulting from the plasmon modes of AgNWs@TiO_2_. Besides, two weak and broad positive absorption peaks appear at lower and higher energies around bleaching plasmon band. The photo-induced absorption wings are consistent with previous reports involving silver nanospheres, gold nanospheres, and gold nanorods^24^, which is attribute to a broadening and blue-shift of the plasmon resonance as electron temperature rises after optical pumping^49^. The relaxation of carriers cooling is investigated by varying the TiO_2_ coating layer thickness. The normalized decay of the photobleaching at 357 nm in Fig. 6c shows that not only does the TiO_2_ coating induce a faster relaxation time as compared to pure AgNWs, but also the thinner the TiO_2_ layer the faster the decay time. The corresponding time constants of carrier lifetimes were best fitted with a mono exponential function and those derived from the mono exponential listed in Table 2 highlight the strong influence of the shell thickness on the downfall decay time from 5.9 ps of τ_e_-ph in AgNWs to 0.87 ps in the AgNWs@TiO_2_ core-shell structure. Increasing the shell thickness leads to a subsequent increase of this life time up to a saturation value for the 20 nm and 50 nm samples of 2.55 ps (inserted curve in Fig. 6c), but still shorter than pure AgNWs.

**Table 2:** Amplitudes (A) and time constants (τ) derived from exponential fit of the transient absorption of various TiO_2_ shell thicknesses at the photobleached band located at 357 nm (349 nm for pure AgNWs).

Sample	Amplitudes (A)	Time constants (τ, ps)
AgNWs	−4.95 ± 0.09	5.9 ± 0.2
AgNWs + TiO_2_ 5 nm	−3.36 ± 0.09	0.87 ± 0.09
AgNWs + TiO_2_ 10 nm	−6.1 ± 0.24	1.87 ± 0.09
AgNWs + TiO_2_ 20 nm	−18.7 ± 0.7	2.53 ± 0.1
AgNWs + TiO_2_ 50 nm	−1.72 ± 0.23	2.55 ± 0.4

The existence of a direct contact between the semiconductor and the metallic nanowires facilitates the carrier transfer between them. The photo-excited surface plasmon can decay through non-radiative channel generating electron-hole pairs as mentioned before. A previously established model was employed to study the size-dependent carrier decay time in silver nanoparticles and nanotriangles as^28^:

$$G\propto {\int }_{0}^{{q}_{D}}{q}^{3}{(\frac{{V}_{q}}{{\varepsilon }_{0,q}})}^{2}dq$$

Where the electron-phonon coupling constant, G, is related to the unscreened electron-phonon interaction potential V_q_ and inversely proportional to the dielectric function ε_0,q_, which is associated to the electron density n_e_. The spill-out of electron changes the ε, making less screening and improving the coupling rate. Hot electron injection could happen from the metal to the semiconductor conduction band to interrupt original electron-phonon coupling channel. The new decay channel through injection may provide similar effect and accelerate the relaxation of hot electrons in silver nanowires. Besides the hot electron transfer, other mechanisms such as defect-mediated recombination and PIRET process could also exist in this nanostructure system leading to shell thickness dependent decay lifetime. However, it is worth noting that it is difficult for our current pump-probe transient set-up to discern these mechanisms and it is apparently beyond our scope in the work.

In this system, the more available hot carriers transferred from the metal they could more efficiently result in a faster energetic charge carrier injection into TiO_2_, leading to a shorter carrier lifetime as shown in TAS measurements which is similar to hot electron injection in MoS_2_ nanosheets^50^. Moreover, the performed absorption and scattering calculations for pure AgNWs and AgNWs with 20 nm TiO_2_ (Fig. S5) show that the scattering is more dominant in AgNWs than in the core-shell while the absorption fraction prevails in the core-shell nanowires. TiO_2_ coatings improve the generation of electron-hole pairs derived from plasmon decay because the radiation pathway is determined by scattering while the non-radiative electron-hole pairs pathway results from absorption. The simulation outcomes are similar to those presented in a recent report on Ag-Pt core-shell nanocubes^51^. The larger production of e-h pairs may strongly increase the carrier injection rate. The simulated electric field enhancement also demonstrated the thinner shell sample exhibiting much larger near-field hot spot enhancement than the thicker one, which is shown in Fig. S6. However, the easy injection from silver to TiO_2_ via overcoming Schottky barrier could also lead to reverse transfer due to the lack of insulator at the silver-TiO_2_ interface. The thicker TiO_2_ layers also create more photo-excited electrons in the conduction band due to the high energy of pump laser. Owing to the interband transition of TiO_2_, this provides more pathways for a back transfer from TiO_2_ to AgNWs, as a result of less hot electron extraction and spill out of electrons. In addition, the thicker shells could result in a slower back recombination rate of injected hot electrons in AgNWs^52^, which also leads to a longer carrier relaxation time for the 357 nm plasmon band. The saturation of the lifetime constant with the TiO_2_ shell thickness (up to 50 nm) is significant of a possible electron transfer equilibrium between the metal and the semiconductor. The final lifetime value still lower than that of the bare individual silver may be attributed to a larger absorption cross-section in plasmonic nanowires. In addition, Govorov’s recent simulation work also demonstrated that the thinner TiO_2_ shell could result in more energetic electrons in hot spots, which provides greater opportunities for hot electron injection processes due to the induced carriers with higher energy^53^. Meanwhile, the hot electrons injection is a competing process against electron-electron scattering, which reduces the excited energy of an electron preventing the excited electron from having enough energy to cross the interfacial energy barrier^54^. Besides, weaker e-e scattering indicates a lower initial temperature of electrons T_e_, leading to a faster e-ph coupling decay time according to the two-temperature models. The higher hot electron injection efficiency could induce faster decay of electron phonon coupling^55^. According to the above-mentioned proposal, we conclude the hot electron transfer process between metal and TiO_2_ could play an important role for their transient dynamics performance.

### Relaxation dynamics after AuNPs decoration

**Figure 7:**
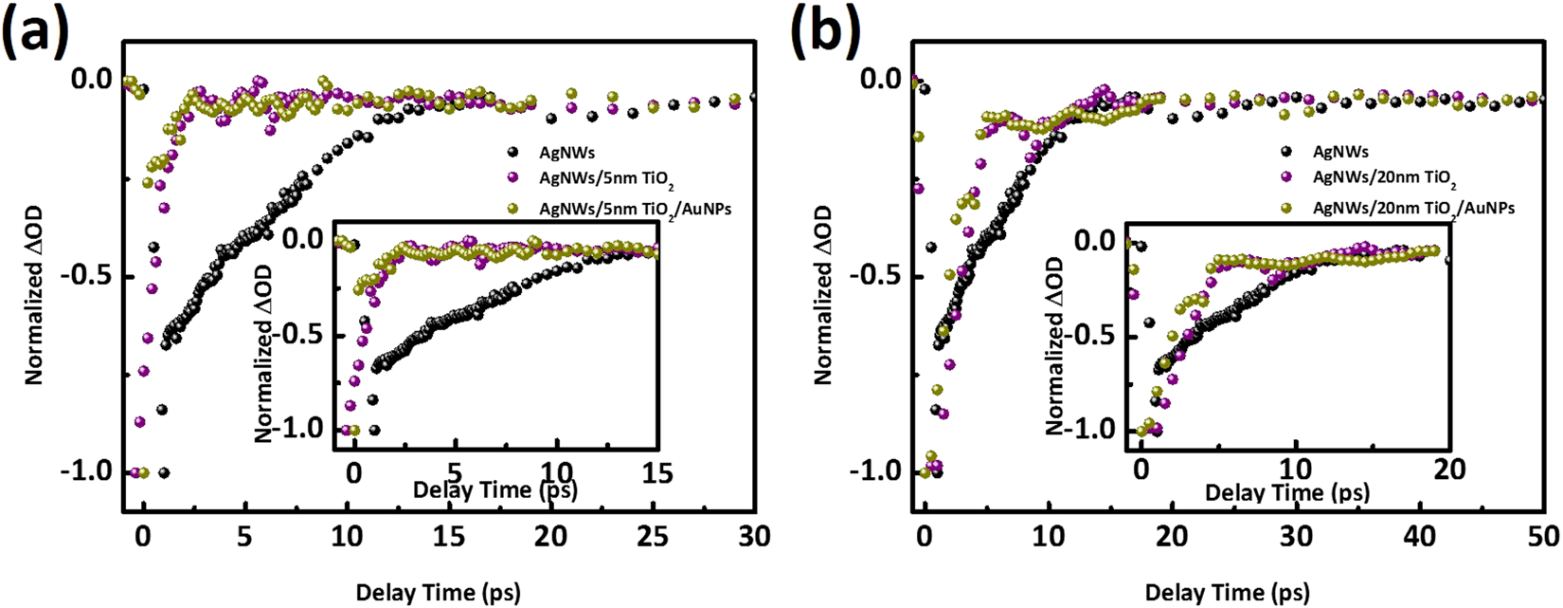
Normalized decay kinetics at 357 nm for the reference AgNWs (black sphere), before (purple) and after (yellow) addition of AuNPs onto AgNWs@TiO_2_, with 5 nm (**a**) and 20 nm (**b**) TiO_2_ shell thickness.

The small APTES-modified AuNPs have been deposited onto AgNWs@TiO_2_ surface to form the desirable nanostructures. The concentration of gold NPs was fixed low enough so as it did not affect their surface plasmon behaviour apparently. The same kind of transient spectroscopic studies were performed after addition of AuNPs on 5 nm TiO_2_ layer (Fig. 7a) and 20 nm TiO_2_ layer (Fig. 7b), and we could easily observe that the bleaching decay for AgNWs plasmon got faster after AuNPs decoration. Table 3 gives a summary of their dynamics performance. With the presence of AuNPs onto the TiO_2_ surface, the lifetime of e-ph coupling decreases to 0.43 ps for the 5 nm thickness TiO_2_, which exhibits a 51% change. Unexpectedly the decrease is of only 7% for the 20 nm TiO_2_ sample, which decreases from 2.53 ps to 2.35 ps after AuNPs decoration. The detailed experimental results are shown in Supplementary Information (Fig. S4).

**Table 3:** Amplitudes (A), time constants (τ), derived from exponential fits of the transient absorption for the AuNPs decorated onto 5 nm and 20 nm TiO_2_ shells at the photobleaching band located at 357 nm.

Sample	Amplitudes (A)	Time constants (τ, ps)
AgNWs + TiO_2_ 5 nm	−3.36 ± 0.09	0.87 ± 0.09
AgNWs + TiO_2_ 5 nm + AuNPs	−6.64 ± 0.03	0.43 ± 0.04
AgNWs + TiO_2_ 20 nm	−18.7 ± 0.7	2.53 ± 0.09
AgNWs + TiO_2_ 20 nm + AuNPs	−28.7 ± 0.9	2.35 ± 0.09

The small AuNPs could bring external plasmon-phonon coupling to assist the original plasmon induced electron-phonon interaction of silver nanowires and electron transfer, which is similar to the perovskites-TiO_2_ injection^56^. The result demonstrates that the fact to have two plasmon modes generates a synergy for a hot electron transfer from silver nanowires and a faster electron injection into TiO_2_. This is confirmed by the role of the shell thickness since the thinner the TiO_2_ layer exhibits the more efficient the bleaching decay. Nevertheless, the detailed mechanism of plasmon-assisted fast bleaching decay still needs to be further investigated. It is also worthwhile noting that the thinner TiO_2_ layer allows a much severe decrease in decay time after the same deposition of AuNPs, highlighting the stronger interaction between AuNPs and AgNWs plasmons because of shorter distance. Such studies of carrier relaxation dynamics provide a promising pathway to design nanostructures with a high efficiency for hot electron injection focusing on energy conversion devices. However, further fundamental studies are needed to clarify these hybrid nanostructures for practical catalysis applications. Among these works there is a need (i) to optimize desirable plasmon nanohybrids through preparation of nanoobjects with distinct contribution of interband/intraband transition under pump wavelength dependent TAS measurement^57^, (ii) to elucidate different plasmon-induced transfer mechanism such as charge transfer or energy transfer according to tunable plasmon/semiconductor spectra overlap^58^, (iii) to determine carrier transfer channels such as sequential process or direct process stem from chemical interface damping^59^ under line width observation^60^. The ultrafast and spatially resolved measurement technique could provide a promising approach to analysis the physical and chemical origins of these photo processes.

## Discussion

In summary, we have prepared different shell thicknesses of TiO_2_ coating onto silver nanowires surface, and functionalized them with small AuNPs to form AgNWs@TiO_2_@AuNPs nanostructures via a facile wet chemical approach before proceeding to the study of their morphology and steady optical properties. The plasmon decay dynamics of silver nanowires were also measured thanks to ultrafast pump-probe technique. Our results indicate a faster decay time after TiO_2_ shell coating and its dependence on the shell thickness, showing that this decay time decreases as the shell thickness becomes thinner. The shorter carrier lifetimes obtained from TAS measurements reveal the strong electron-phonon interaction, which could be a result of a hot carrier injection process. The simulation of the absorption and the scattering fraction further supports the hypothesis that TiO_2_ coating facilitates the electron-hole pairs formation. Lifetimes obtained from photobleach recovery measurements suggest that the presence of gold nanoparticles did improve the hot carrier transfer via plasmonic coupling effects and the emerging transfer channel is greatly affected by the TiO_2_ shell thickness. We expect these multiple plasmon modes of such core-shell nanostructures to be a potential tool for enhancing the efficiencies of photo-driven energy conversion devices.

## Methods

### Synthesis of AgNWs and surface modification

The PVP capped AgNWs were prepared by a modified polyol method^61^. The synthesis yielded a large amount of silver nanowires with a typical diameter of 40 ± 2.1 nm and lengths of tens of µm with narrow size distribution. Then, these AgNWs were surface modified with mercaptoundecanoic acid (MUA) as follows: 1 mL of as-synthesized AgNWs (0.1 mg/mL) was added with 18 mL pure ethanol and mixed with 1 mL of a 10 mM MUA solution in pure ethanol. After that, the mixture was kept on a roller-mixer overnight at a rate of 250 rpm/min. Then, the mixture was washed with pure ethanol and centrifuged 3 times before eventually keeping them in 20 mL pure ethanol at a concentration of 0.08 mg/mL.

### Synthesis of APTES-AuNPs

Around 4 nm APTES-AuNPs were synthesized by using X. Sun’s method with slightly modifications^62^. Typically, 400 μL of APTES were added to 0.84 mL of a 0.1 M HAuCl_4_ aqueous solution with 2.66 mL Milli-Q water under vigorous stirring at room temperature to obtain supramolecular microstructures of the mixture. Then the colloidal solution was heated up to 100 °C for 30 min and kept at room temperature overnight, followed by centrifugation to remove free APTES and other by-products. The synthesized AuNPs colloid has a concentration of 10.88 × 10^3^ nM and an average diameter of 4.2 nm as observed by TEM measurement (not shown).

### Controlled deposition of TiO_2_ on AgNWs

The deposition of TiO_2_ on the AgNWs surface was performed using acetylacetone as a retardant reagent to control the hydrolysis and polycondensation of the titanium precursor. In an optimized synthesis, a total 23 mL solution including: 20 mL of as-synthesized MUA functionalized AgNWs (0.08 mg/ml) in pure ethanol and a mixture of 0.25 mL of Milli-Q water and 2.75 mL absolute ethanol were introduced in a 50 mL flat-bottom flask with stirring as a template solution. In parallel, 1 mL of precursor solution at a concentration of 14 mM TTIP mixed with Acac (1:2 molar ratio) was prepared. The precursor solution was then diluted with various fractions of pure ethanol. The thickness of the TiO_2_ coating was effectively dependent on the degree of dilution, typically to obtain 5, 10, 20, 50 and 70 nm thickness of TiO_2_, the precursor solution must be diluted 10, 5, 4, 3 and 2 times respectively via addition of pure ethanol. 1 mL of either of these light yellow solutions was then injected into the template solution at a rate of 1 mL/h by a syringe pump. After the completion of the injection, the resulting colloidal suspension was heated up to 80 °C with stirring for 1.5 h to ensure the condensation procedure was completed. The colloidal suspension was then washed by 5 extensive centrifugation cycles with pure EtOH and eventually stored in 25 mL pure ethanol at a concentration of approximately 0.5, 0.8, 1.4, 1.9 and 2.1 mg/mL for ca. 5, 10, 20, 50 and 70 nm samples respectively. The obtained colloidal suspensions hereafter named as AgNWs@TiO_2_ are then ready for the next step. In any case, for large production, the reaction can simply be scaled up to 10–20 times.

### Synthesis of AgNWs@TiO_2_@AuNPs

Typically, for AgNWs with 5 nm TiO_2_ shell, 0.1 mL, 10.88 × 10^3^ nM of the as-synthesized APTES-AuNPs was diluted ten times with 0.9 mL pure water and sonicated for 10 min before use. Then 1 mL of 0.5 mg/mL AgNWs@TiO_2_ was added to the APTES-AuNPs solution with 15 min sonication. The mixture was kept at room temperature on a roller-mixer overnight at a rate of 250 rpm/min. Then, the mixture was washed 3 times with pure water and precipitated by centrifugation and kept eventually in 2 mL pure water at a concentration of 0.1 mg/mL. The obtained colloidal suspension hereafter named as AgNWs@TiO_2_@AuNPs is then ready for the next step. In any case, for large production, the reaction can be simply scaled up to 10–20 times.

### Morphology characterization

Transmission electron microscope experiments were performed with a JEOL JEM-1400Plus microscope operating at 120 kV. The samples were prepared as follows: colloids were diluted in ethanol and one drop of the diluted suspension was deposited on a copper grid coated with a carbon membrane. Chemical analysis was carried out by STEM coupled to EDX were acquired with a JEOL 2200 FS equipped with a field emissive gun, operating at 200 kV and with a point resolution of 0.23 nm.

### Optical simulation

The simulated extinction spectra were performed using a three-dimensional module on Finite-difference time-domain (FDTD) based software (Lumerical Solutions). The model systems include a background surrounding with water and the dielectric function data for silver taken from Johnson and Christy.

### Visible femtosecond transient absorption measurements

In this study Spectra-Physics Solstice were utilized as the ultrafast laser beams with a regeneratively amplified Ti: sapphire laser system (Coherent Legend, 800 nm, 100 fs and 1 kHz repetition rate) and the signal acquisition was collected by optical fiber. The 800 nm output laser beams were firstly split at BS1, one part was through into Traveling Wave Optical Parametric Amplifier (TOPAS) define to generate tunable pulse for pump. A series of neutral-density filter wheels were used to adjust the power of the pump beam. The pump beam was focused at the sample with a beam waist of about 200 μm as following chopped by as synchronized chopper to 500 Hz. Another part from beam split was focusing the 800 nm probe, which went into a CaF_2_ window to create a white light continuum (WLC) from 350 nm to 800 nm. The probe beams were focused into a fiber-coupled multichannel spectrometer with complementary metal-oxide-semiconductor (CMOS) sensors and detected at a frequency of 1 kHz. The delay between the pump and probe pulses was controlled by a motorized delay stage. The samples were dispersed in ethanol and averaged over several spots to ensure uniformity. The collected data are fitted in Origin 8.5 software with proper exponential fittings with respect that the time range presented are as representative as possible.

## Electronic supplementary material

Supporting Information

## References

[1] Mubeen S (2013). An autonomous photosynthetic device in which all charge carriers derive from surface plasmons. *Nat*. Nanotech..

[2] Christopher P, Xin H, Linic S (2011). Visible-light-enhanced catalytic oxidation reactions on plasmonic silver nanostructures. *Nat*. Chem..

[3] Sousa-Castillo A (2016). Boosting Hot Electron-Driven Photocatalysis through Anisotropic Plasmonic Nanoparticles with Hot Spots in Au–TiO_2_ Nanoarchitectures. J. Phys. Chem. C.

[4] Knight MW, Sobhani H, Nordlander P, Halas NJ (2011). Photodetection with Active Optical Antennas. Science.

[5] Atwater HA, Polman A (2010). Plasmonics for improved photovoltaic devices. Nat. Mater..

[6] Gu M, Li X, Cao Y (2014). Optical storage arrays: a perspective for future big data storage. Light Sci Appl.

[7] Mansuripur M (2009). Plasmonic nano-structures for optical data storage. Opt. Express.

[8] Clavero C (2014). Plasmon-induced hot-electron generation at nanoparticle/metal-oxide interfaces for photovoltaic and photocatalytic devices. Nat. Photon..

[9] Moskovits M (2015). The case for plasmon-derived hot carrier devices. Nat. Nano..

[10] Linic S, Christopher P, Ingram DB (2011). Plasmonic-metal nanostructures for efficient conversion of solar to chemical energy. Nat. Mater..

[11] Brongersma ML, Halas NJ, Nordlander P (2015). Plasmon-induced hot carrier science and technology. *Nat*. Nano..

[12] Kale MJ, Avanesian T, Christopher P (2014). Direct Photocatalysis by Plasmonic Nanostructures. ACS Catal..

[13] Govorov AO, Zhang H, Gun’ko YK (2013). Theory of Photoinjection of Hot Plasmonic Carriers from Metal Nanostructures into Semiconductors and Surface Molecules. J. Phys. Chem. C.

[14] Manjavacas A, Liu JG, Kulkarni V, Nordlander P (2014). Plasmon-Induced Hot Carriers in Metallic Nanoparticles. ACS Nano.

[15] Odom TW, Schatz GC (2011). Introduction to Plasmonics. Chem. Rev..

[16] Linic S, Aslam U, Boerigter C, Morabito M (2015). Photochemical transformations on plasmonic metal nanoparticles. Nat. Mater..

[17] Hartland GV, Besteiro LV, Johns P, Govorov AO (2017). What’s so Hot about Electrons in Metal Nanoparticles?. ACS Energy Lett..

[18] Mongin D (2012). Ultrafast Photoinduced Charge Separation in Metal–Semiconductor Nanohybrids. ACS Nano.

[19] Erwin WR, Zarick HF, Talbert EM, Bardhan R (2016). Light trapping in mesoporous solar cells with plasmonic nanostructures. Energ. Environ. Sci..

[20] Furube A, Du L, Hara K, Katoh R, Tachiya M (2007). Ultrafast Plasmon-Induced Electron Transfer from Gold Nanodots into TiO_2_ Nanoparticles. J. Am. Chem. Soc..

[21] Hyun B-R (2008). Electron Injection from Colloidal PbS Quantum Dots into Titanium Dioxide Nanoparticles. ACS Nano.

[22] Del Fatti N (2000). Nonequilibrium electron dynamics in noble metals. Phys. Rev. B.

[23] Christophe Voisin NDF, Dimitris Christofilos A, Vallée F (2001). Ultrafast Electron Dynamics and Optical Nonlinearities in Metal Nanoparticles. J. Phys. Chem. B.

[24] Link S, El-Sayed MA (1999). Spectral Properties and Relaxation Dynamics of Surface Plasmon Electronic Oscillations in Gold and Silver Nanodots and Nanorods. J. Phys. Chem. B.

[25] Kamat PVP (2002). Photochemical and Photocatalytic Aspects of Metal Nanoparticles. J. Phys. Chem. B.

[26] Dacosta Fernandes B (2013). Electron–Phonon Scattering in 2D Silver Nanotriangles. J. Phys. Chem. C.

[27] Hartland GV (2011). Optical Studies of Dynamics in Noble Metal Nanostructures. Chem. Rev..

[28] Arbouet A (2003). Electron-Phonon Scattering in Metal Clusters. Phy. Rev. Lett..

[29] Guo P, Schaller RD, Ketterson JB, Chang RPH (2016). Ultrafast switching of tunable infrared plasmons in indium tin oxide nanorod arrays with large absolute amplitude. Nat. Photon.

[30] Diroll BT, Guo P, Chang RPH, Schaller RD (2016). Large Transient Optical Modulation of Epsilon-Near-Zero Colloidal Nanocrystals. ACS Nano.

[31] Yang X (2017). Plasmon-exciton coupling of monolayer MoS_2_-Ag nanoparticles hybrids for surface catalytic reaction. Mater Today Energy.

[32] Lin W (2017). Physical mechanism on exciton-plasmon coupling revealed by femtosecond pump-probe transient absorption spectroscopy. Mater Today Phys.

[33] Lin W, Cao Y, Wang P, Sun M (2017). Unified Treatment for Plasmon–Exciton Co-driven Reduction and Oxidation Reactions. Langmuir.

[34] Cao E (2017). Electrooptical Synergy on Plasmon–Exciton-Codriven Surface Reduction Reactions. Adv. Mater. Interfaces.

[35] Ding Q (2016). Ultrafast Dynamics of Plasmon-Exciton Interaction of Ag Nanowire- Graphene Hybrids for Surface Catalytic Reactions. Sci. Rep..

[36] Cushing SK (2012). Photocatalytic Activity Enhanced by Plasmonic Resonant Energy Transfer from Metal to Semiconductor. J. Am. Chem. Soc..

[37] Li J (2013). Ag@Cu2O Core-Shell Nanoparticles as Visible-Light Plasmonic Photocatalysts. ACS Catal..

[38] Li J (2015). Plasmon-induced resonance energy transfer for solar energy conversion. Nat. Photon..

[39] You JH, Kuo YY, Hsu KY (2015). Influence of Various Reaction Parameters on the Process for Preparation of SiO_2_/TiO_2_ Core-Shell Particles. J. Nano. Res..

[40] Bian RX, Dunn RC, Xie XS, Leung PT (1995). Single Molecule Emission Characteristics in Near-Field Microscopy. Phys. Rev. Lett..

[41] Johnson PB, Christy RW (1972). Optical Constants of the NobleMetals. Phy. Rev. B..

[42] Sun Y, Mayers B, Herricks T, Xia Y (2003). Polyol Synthesis of Uniform Silver Nanowires:  A Plausible Growth Mechanism and the Supporting Evidence. Nano Lett..

[43] Zhang J (2003). Ultrasonication-Induced Formation of Silver Nanofibers in Reverse Micelles and Small-Angle X-ray Scattering Studies. J. Phys. Chem. B.

[44] Hu H, Pauly M, Felix O, Decher G (2017). Spray-assisted alignment of Layer-by-Layer assembled silver nanowires: a general approach for the preparation of highly anisotropic nano-composite films. Nanoscale.

[45] Zhenghua W, Jianwei L, Xiangying C, Junxi W, Yitai Q (2005). A Simple Hydrothermal Route to Large‐Scale Synthesis of Uniform Silver Nanowires. Chem. Eur. J.

[46] da Silva RR (2016). Facile Synthesis of Sub-20 nm Silver Nanowires through a Bromide-Mediated Polyol Method. ACS Nano.

[47] Groeneveld RHM, Sprik R, Lagendijk A (1995). Femtosecond spectroscopy of electron-electron and electron-phonon energy relaxation in Ag and Au. Phys. Rev. B.

[48] Grennell AN, Utterback JK, Pearce OM, Wilker MB, Dukovic G (2017). Relationships between Exciton Dissociation and Slow Recombination within ZnSe/CdS and CdSe/CdS Dot-in-Rod Heterostructures. Nano Lett..

[49] Brown AM (2017). Experimental and Ab Initio Ultrafast Carrier Dynamics in Plasmonic Nanoparticles. Phy. Rev. Lett..

[50] Wang L (2017). Slow cooling and efficient extraction of C-exciton hot carriers in MoS2 monolayer. Nat. Commun..

[51] Aslam U, Chavez S, Linic S (2017). Controlling energy flow in multimetallic nanostructures for plasmonic catalysis. Nat. Nano..

[52] Karam TE, Khoury RA, Haber LH (2016). Excited-state dynamics of size-dependent colloidal TiO_2_-Au nanocomposites. J. Chem. Phys..

[53] Harutyunyan H (2015). Anomalous ultrafast dynamics of hot plasmonic electrons in nanostructures with hot spots. Nat. Nano..

[54] Wu K, Rodríguez-Córdoba WE, Yang Y, Lian T (2013). Plasmon-Induced Hot Electron Transfer from the Au Tip to CdS Rod in CdS-Au Nanoheterostructures. Nano Lett..

[55] Ratchford DC, Dunkelberger AD, Vurgaftman I, Owrutsky JC, Pehrsson PE (2017). Quantification of Efficient Plasmonic Hot-Electron Injection in Gold Nanoparticle–TiO_2_ Films. Nano Lett..

[56] Zarick HF (2017). Ultrafast carrier dynamics in bimetallic nanostructure-enhanced methylammonium lead bromide perovskites. Nanoscale.

[57] Schlather AE (2017). Hot Hole Photoelectrochemistry on Au@SiO_2_@Au Nanoparticles. J. Phys. Chem. Lett..

[58] Li J (2014). Solar Hydrogen Generation by a CdS-Au-TiO_2_ Sandwich Nanorod Array Enhanced with Au Nanoparticle as Electron Relay and Plasmonic Photosensitizer. J. Am. Chem. Soc..

[59] Wu K, Chen J, McBride JR, Lian T (2015). Efficient hot-electron transfer by a plasmon-induced interfacial charge-transfer transition. Science.

[60] Hoggard A (2013). Using the Plasmon Linewidth To Calculate the Time and Efficiency of Electron Transfer between Gold Nanorods and Graphene. ACS Nano.

[61] Sun Y, Yin Y, Mayers BT, Herricks T, Xia Y (2002). Uniform Silver Nanowires Synthesis by Reducing AgNO_3_ with Ethylene Glycol in the Presence of Seeds and Poly(Vinyl Pyrrolidone). Chem. Mater..

[62] Sun X, Wei W (2010). Electrostatic-Assembly-Driven Formation of Micrometer-Scale Supramolecular Sheets of (3-Aminopropyl)triethoxysilane(APTES)-HAuCl_4_ and Their Subsequent Transformation into Stable APTES Bilayer-Capped Gold Nanoparticles through a Thermal Process. Langmuir.

