# Supplementary material for: Plasmon-induced hot electron transfer in AgNW@TiO2@AuNPs nanostructures
Source: Sci Rep. 2018 Sep 20;8:14136. doi: 10.1038/s41598-018-32510-2 (PMC6148267; doi:10.1038/s41598-018-32510-2)
Supplement: Supplementary file 1 — Supporting Information [file 41598_2018_32510_MOESM1_ESM.docx]

**Electronic Supplementary Information**

**Plasmon-induced hot electron transfer in AgNW@TiO_2_@AuNPs nanostructures**

Jiaji Cheng,^1,2^ Yiwen Li,^3,4^ Marie Plissonneau,^1^ Jiagen Li,^5^ Junzi Li,^2^Rui Chen,^3^ Zikang Tang,^4^ Lauriane Pautrot-d'Alençon,^6^ Tingchao He,^2^* Mona Tréguer-Delapierre^1^* and Marie-Hélène Delville^1^*

^1^*CNRS, Univ. Bordeaux, ICMCB, UMR 5026, F-33608, Pessac, France.*

*^2^College of Physics and Energy, Shenzhen University, Shenzhen 518060, People’s Republic of China.*

*^3^Department of Electrical and Electronic Engineering, Southern University of Science and Technology, Shenzhen, 518055, China.*

*^4^The Institute of Applied Physics and Materials Engineering, University of Macau, Avenida da Universidade, Taipa, Macau, China*

*^5^School of Science and Engineering, The Chinese University of Hong Kong, Shenzhen, People’s Republic of China*

*^6^Solvay, 52 rue de la Haie Coq, Aubervilliers F93308, France.*

Corresponding author: marie-helene.delville@icmcb.cnrs.fr; mona.treguer-delapierre@icmcb.cnrs.fr; tche@szu.edu.cn

Figure S1. Transient Absorption Spectrometer System.

Figure S2. (a) and (b), TEM images of AgNWs@TiO_2_@AuNPs nanostructures with (a) 10 nm and (b) 20 nm TiO_2_thickness. Inserted are magnified images. (c) Corresponding normalized UV spectra of as-synthesized AgNWs@TiO_2_@AuNPs nanostructures with different TiO_2_ thicknesses. The black dashed line at 538 nm indicates the small absorption peak in the case of AgNWs@TiO_2_@AuNPs with 5 nm and 10 nm thickness of TiO_2_. When the thickness of TiO2 is 20 nm, the absorption peak is hardly visible probably lost in the tail of the red shifted transverse mode of the AgNWs.

Figure S3. Positive Gold nanoparticles with APTES modified (a). TEM image of 4.1 nm AuNPs (b). Absorption spectrum of AuNPs (c).

Figure S4. ΔOD spectral map of AgNW (a) and AgNW/TiO_2_/AuNP with 5 nm (b) and 20 nm (c) TiO_2_ shell. (d-f) Photobleach decay for the same samples of (a)-(c) respectively.

Figure S5. Calculated fraction of absorption and scattering of pure AgNWs (a) and AgNWs/TiO_2_ with 20 nm shell thickness (b).

Figure S6. Field enhancement calculations for AgNW/TiO_2_/GNPs with 5 (a), 10 (b), 20 (c) and 50 nm (d) TiO_2_ thickness. Right Y axis is the electric field enhancement E/E_0_.
